# Circular RNA circ_0008305 aggravates hepatocellular carcinoma growth through binding to miR‐186 and inducing TMED2

**DOI:** 10.1111/jcmm.15945

**Published:** 2020-11-18

**Authors:** Xiaoyu Zhang, Hui‐Hui Hao, Hai‐Wen Zhuang, Jian Wang, Yu Sheng, Fang Xu, Jin Dou, Chuang Chen, Yang Shen

**Affiliations:** ^1^ Division of Gastrointestinal Surgery Huai'an Second People's Hospital and The Affiliated Huai'an Hospital of Xuzhou Medical University Huai'an China; ^2^ Department of Pharmacology Jiangsu College of Nursing Huai'an China; ^3^ Division of Hepatobiliary Surgery Huai'an Second People's Hospital and The Affiliated Huai'an Hospital of Xuzhou Medical University Huai'an China; ^4^ Operating Room Huai'an Second People's Hospital and The Affiliated Huai'an Hospital of Xuzhou Medical University Huai'an China

**Keywords:** circ_0008305, hepatocellular carcinoma, miR‐186, TMED2

## Abstract

Dysregulation of circRNAs is reported to exert crucial roles in cancers, including hepatocellular carcinoma (HCC). So far, the function of circRNAs in HCC development remains poorly known. Currently, our data showed that circ_0008305 was highly elevated in HCC cell lines and 30 paired tissue samples of HCC. As evidenced, suppression of circ_0008305 repressed HCC cell growth significantly. Meanwhile, up‐regulation of circ_0008305 significantly reduced HCC cell growth. Mechanistically, we displayed that circ_0008305 could bind with miR‐186 by using bioinformatics analysis. miR‐186 has been reported to be a crucial tumour oncogene in many cancers. In addition, we proved miR‐186 was greatly decreased in HCC. The direct correlation between miR‐186 and circ_0008305 was confirmed in our work. In addition, up‐regulation of miR‐186 obviously restrained HCC progression. Increased expression of transmembrane p24 trafficking protein 2 (TMED2) is significantly related to the unfavourable outcomes in cancer patients. At our present work, we proved that TMED2 could act as a direct target of miR‐186. Mechanistically, we demonstrated that circ_0008305 up‐regulated TMED2 expression by sponging miR‐186, which resulted in significantly induced HCC progression in vitro and in vivo. These revealed the significant role of circ_0008305 in HCC progression, which might indicate a new perspective on circRNAs in HCC development.

## 1 INTRODUCTION

Hepatocellular carcinoma (HCC) is a prevalent cancer, and it is a leading cause of cancer‐related deaths across the world.^1^ Meanwhile, HCC results in a great number of cancer‐related death.^2^ The main therapy for HCC includes surgery resection and chemotherapy. During past several decades, although advanced advances are made in the treatment of HCC, prognosis of HCC is very poor with 5‐year survival rate less than 25%.^3^ Therefore, it is significant to identify effective biomarkers for HCC and develop therapeutic targets.

In recent years, circRNAs have obtained increasing attention. They are a new class of non‐coding RNAs with little potential of protein‐coding humans.^4^, ^5^, ^6^ As well reported, circRNAs are more stable compared with the linear RNAs.^7^ CircRNAs contain covalently closed loop structures with no 5′ to 3′ polarity or a polyadenylated tail.^8^, ^9^ Accumulating evidence has reported circRNAs exhibit crucial roles in tumour biology through regulating diverse mechanisms.^10^ For instance, in gastric cancer, circ_100269 is decreased and it can repress tumour development through sponging miR‐630.^11^ In bladder cancer, Cdr1as can exert a tumour inhibitory function by sponging miR‐135a.^12^ In addition, circ_100876 can induce breast cancer progression through sponging miR‐361‐3p.^13^ CircRHOT1 induces HCC progression through the initiation of NR2F6.^14^

Here, we concentrated on the effects of circ_0008305 in HCC and we reported decrease of circ_0008305 inhibited cell proliferation, migration and invasion. Additionally, it was demonstrated that circ_0008305 may sponge miR‐186 to increase expression of TMED2 in HCC.

## 2 MATERIALS AND METHODS

### 2.1 Patient samples

This work was approved by Medical Ethics Committee of Huai'an Second People's Hospital and The Affiliated Huai'an Hospital of Xuzhou Medical University. 60 pairs of surgical HCC and adjacent non‐tumour specimens from February 2016 to February 2018 were collected. The patients all signed the informed consents. The tissue samples were immediately frozen during the operation at −80°C.

### 2.2 Cell culture

SK‐HEP‐1, Huh‐7, Hep‐3B, HepG2, SMMCC7721 and LO2 cells were obtained from ATCC (Manassas, VA, USA). Cells were incubated in DMEM medium with 20% FBS, 2 mmol/L l‐glutamine, 100 units/mL of penicillin and 100 µg/mL of streptomycin (Gibco, Aukland, New Zealand) in an incubator with 5% CO_2_ at 37°C.

### 2.3 Cell transfection and transduction

The full‐length of circ_0008305 was sub‐cloned into the lentivirus vector (LV‐ circ_0008305) by GeneChem (Shanghai, China). The lentivirus vector with shRNA sequence for circ_0008305 or sh‐NC was cloned by GeneChem (Shanghai, China). miR‐186 mimics, inhibitors. TMED2 siRNA and their negative controls were purchased from Hanheng Biotech (Shanghai, China). Cell transfection was performed using Lipofectamine 3000 (Invitrogen).

### 2.4 Cell Counting Kit‐8 assay

To test cell survival, 3 × 10^3^ cells were seeded into 96‐well plates. Cell Counting Kit‐8 (CCK‐8) assay was obtained from Dojindo Laboratories (Kumamoto, Japan) and carried out. OD values at 450 nm were measured at indicated time point using a SpectraMax microtiter plate reader.

### 2.5 Colony formation assay

To carry out cell colony formation experiment, 600 cells were grown into the 6‐well plates and routinely cultured for two weeks. Then, cells were fixed using 30% formaldehyde for 15 minutes and stained using 0.1% crystal violet (Beyotime Biotechnology, Shanghai, China). Colonies numbers were assessed by an optical microscope (Nikon).

### 2.6 EdU assay

Cell‐LightTM EdU Kit (RIBOBIO, Guangzhou, China) was carried out to determine the cell proliferation. Images of cells were taken using a fluorescence microscope (Nikon).

### 2.7 Apoptosis analysis

FITC‐Annexin V Apoptosis Detection Kit (KeyGen Biotech, Nanjing, China) was performed to evaluate cell apoptosis. Briefly, 1 × 10^5 ^cells were washed using cold PBS. Cells were resuspended in 100 μL 1× Binding Buffer and incubated with 5 µL Annexin V and 5 µL PI staining solution for 10 minutes. Flow cytometry (BD Biosciences) was carried out within 1 hour.

### 2.8 Wound healing assay

Cells were grown into 6‐well plates each well. Then, a wound was made using a 200 μL pipette tip on the cell monolayer. Then, cell photographs were obtained to assess the area occupied by migratory cells.

### 2.9 Transwell assay

Cell migration and invasion were conducted using transwell chamber (Corning Incorporated). 2 × 10^4^ cells were seeded into upper chamber coated with Matrigel matrix in 200 μL serum‐free DMEM to do invasion analysis. Then, the lower chamber was added with 600 μL complete medium. Then, after incubated for 48 hours, we removed the cells on the upper chamber. The cells in the down chamber were stained using 0.5% crystal violet. A light microscope was used to count the cell number.

### 2.10 Western blotting analysis

Cells were lysed using ice‐cold RIPA lysis buffer containing 1% PMSF (Beyotime). Protein concentration was calculated using a Pierce BCA protein assay kit (Thermo Scientific). TMED2 protein was analysed using an anti‐human TMED2 antibody (Cell Signaling Technology). The protein levels were normalized using GAPDH antibody (Cell Signaling Technology). The protein bonds were visualized using a chemiluminescent detection system (Millipore).

### 2.11 qRT‐PCR

Total RNA was isolated using Trizol reagent. Reverse transcription was carried out using TaKaRa system. Then, real‐time PCR was carried out using on LightCycler 96 (Roche). The expression of target genes was based on the formula 2^^‐∆∆Ct^. Primers were exhibited in Table 1.

**TABLE 1:** Primers for real‐time PCR

Genes	Forward (5′‐3′)	Reverse (5′‐3′)
GAPDH	GGAGATTGTTGCCATCAACG	TTGGTGGTGCAGGATGCATT
Circ_0008305	CGGGCTTTGCCATCAATACC	TTGGCCTTGACAGAATCCAG
miR‐186 TMED2 U6	AAGAATTCTCCTTTTGGGCT CGGACAACAGGAGTACATGGAAGTCCG CTCGCTTCGGCAGCACA	GTGCGTGTCGTGGAGTCG GACCAAAGGACCACTCTGCTGT AACGCTTCACGAATTTGCGT

### 2.12 RNA Pull‐down assay

The biotinylated probe was designed to bind to circ_0008305 with the oligo probe as a control. Circ_0008305 probe (Tsingke) was incubated with streptavidin magnetic beads (Life Technologies). Then, cell lysates were incubated with probe‐coated beads for a whole night. After the beads were washed, the bound miRNAs were extracted by Trizol reagent and subjected to qRT‐PCR assay.

### 2.13 Luciferase activity assay

After cells were seeded in 96‐well plates, cells were cotransfected with a mixture of 50 ng luciferase reporter vectors, 5 ng Renila luciferase reporter vectors and miR‐186 mimics/inhibitors/LV‐circ_0008305/sh‐circ_0008305 at the indicated concentration. 48 hours later, the luciferase activity was tested with a dual‐luciferase reporter assay system (Promega).

### 2.14 Tumour xenografts

Four‐week‐old female nude mice were obtained from the Model Animal Research Center at Nanjing University. Mice were randomly divided into two groups and subcutaneously injected with Huh‐7 cells (5 × 10^6^ cells each mouse, six mice in each group) transfected with LV‐circ_0008305 or sh‐circ_0008305. Calliper was used to measure the length and width of tumour, and the volumes were calculated using: length × width × width/2. After six weeks, mice were killed. All animal experiments were based on the Guide for the Care and Use of Laboratory Animals of the National Institutes of Health.

### 2.15 Immunohistochemistry

Immunohistochemical staining was conducted on 4‐μm‐thick sections. Tumour sections were subjected to deparaffinize and rehydrate with gradient ethanol solutions. After being immersed in antigen retrieval solution and heated, sections were incubated with anti‐human Ki‐67 antibody (Dako) for 1 hour and counterstained with haematoxylin (BASO, China) for 2 minutes.

### 2.16 Statistical analysis

Statistical analysis was conducted using SPSS software (version 22.0, SPSS Inc) or GraphPad Prism 6 (GraphPad). Statistical significance evaluated using Student's *t* test or the ANOVA test. *P* < .05 was considered to be statistically significant.

## 3 RESULTS

### 3.1 Increase of circ_0008305 in HCC

**FIGURE 1:**
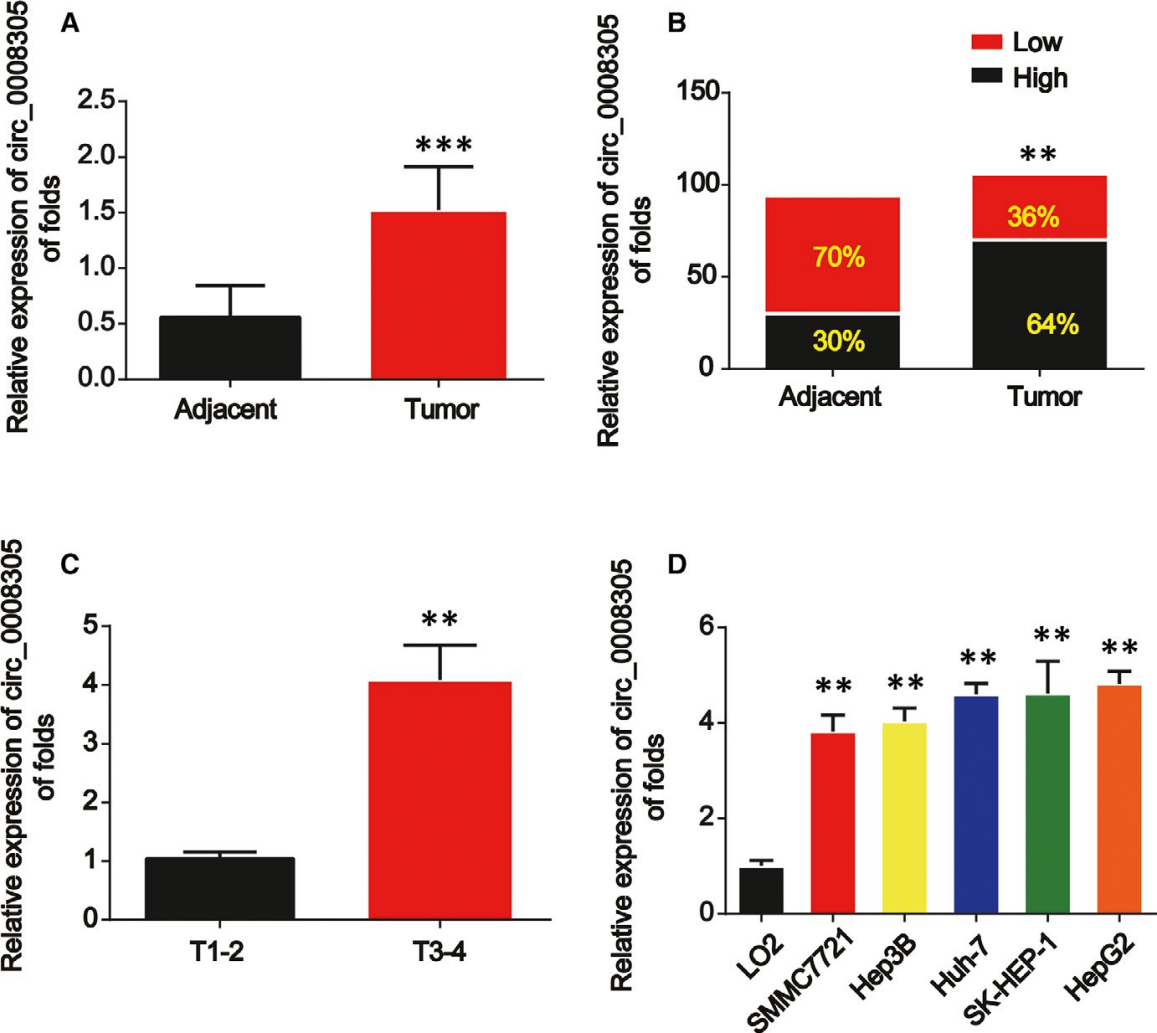
Circ_0008305 were up‐regulated in HCC. A, The expression of circ_0008305 was detected by real‐time PCR in HCC carcinoma and normal adjacent tissues. GAPDH was used as internal control. B, Comparison of low and high expression of circ_0008305 in HCC. C, Expression of circ_0008305 in HCC tissues at different stages. D, The expression level of circ_0008305 in HCC cells (SK‐HEP‐1, Huh‐7, Hep‐3B, HepG2 and SMMCC7721) and LO2 cells. Three independent experiments were carried out. Error bars stand for the mean ± SD of at least triplicate assays. **P < .01; ***P < .001

Firstly, we determined the level of circ_0008305 in HCC tissue samples and HCC cells. After carrying out qRT‐PCR assay, we found that circ_0008305 expression level was obviously up‐regulated in HCC tissues compared with the paired normal tissues as shown in Figure 1A. In HCC tissues, circ_0008305 expression was mostly highly overexpressed than in the paired normal tissues in Figure 1B. Next, in Figure 1C, circ_0008305 expression was significantly elevated in the HCC samples at T3‐T4 stage than in the HCC tissues at an early stage. Then, consistently, in Figure 1D, we observed that circ_0008305 was greatly increased in HCC cells (SK‐HEP‐1, Huh‐7, Hep‐3B, HepG2, SMMC7721) compared with normal LO2 cells. Clinicopathological information of all the patients was displayed in Table 2. High circ_0008305 level was significantly correlated with tumour size, tumour differentiation, TNM stage and metastasis.

**TABLE 2:** The relationship between the expression of circ_0008305 and the clinical characteristics of HCC patients

Parameters	Patient number	Circ_0008305		*P* value
		Low	High	
		expression (<median)	expression (≥ median)	
All cases	60	30	30	
Age, years				
<60	33	16	17	.435
≥60	27	12	15	
Gender				
Male	36	21	15	.589
Female	24	17	7	
Differentiation grade				
Well	22	13	9	**.021**
Moderate	21	15	6	
Poorly	17	6	11	
Tumour size (cm)				
≤5	34	19	15	**.003**
>5	26	14	12	
Tumour capsular				
Incomplete	24	9	15	**.016**
Complete	36	12	24	
TNM stage (I:II:III)				
I	8	3	5	**.001**
II	10	6	4	
III	42	12	30	
Metastasis				
Yes	22	5	17	**.000**
No	48	12	36	

Note

Data from 60 pairs tissue samples of HCC patients were analysed. The median expression level of circ_0008305 served as the cut‐off.

Numbers in bold indicate statistically significant.

### 3.2 Loss of circ_0008305 inhibited HCC cell growth, migration and invasion

**FIGURE 2:**
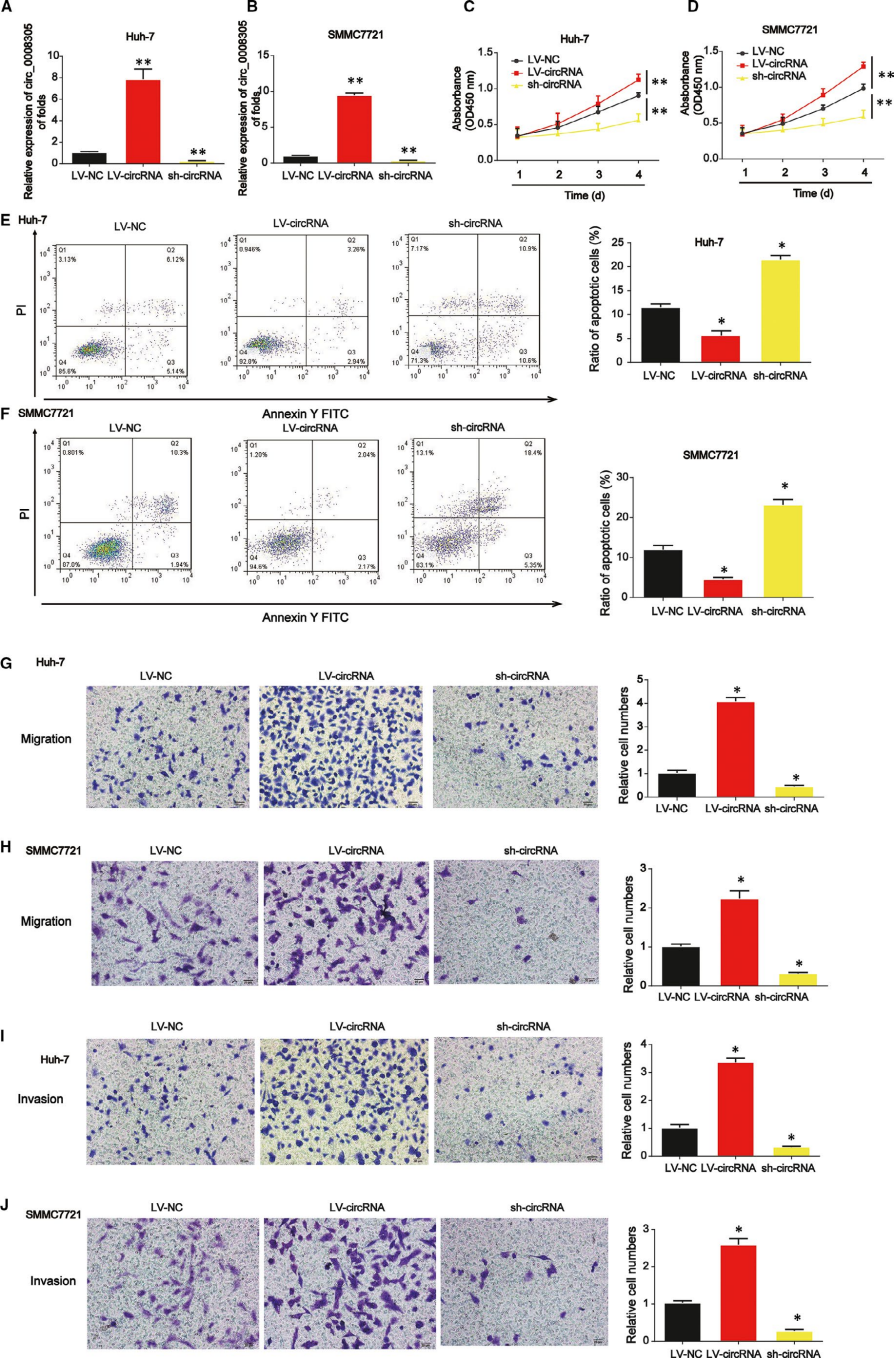
Effects of circ_0008305 siRNA on HCC cell proliferation, migration and invasion. A and B, Expression of circ_0008305 in HCC cells. Cells were infected with LV‐circ_0008305 or circ_0008305 shRNA. C and D, CCK‐8 assay was carried out to test cell viability. E and F, Effects of circ_0008305 on HCC cell apoptosis. Flow cytometry assay was used to detect cell apoptosis. G and H, Effects of circ_0008305 on HCC cell migration. Transwell migration assay was carried out to detect cell migration capacity. I and J, Effects of circ_0008305 on HCC cell invasion. Transwell invasion assay was used to detect cell invasion capacity. Three independent experiments were carried out. Error bars stand for the mean ± SD of at least triplicate assays. *P < .05; **P < .01

Then, we explored the effects of circ_0008305 on HCC cell proliferation. We proved that circ_0008305 was significantly decreased by circ_0008305 shRNA while greatly increased by LV‐circ_0008305 in Huh‐7 and SMMC7721 cells as demonstrated in Figure 2A,B. In Figure 2C,D, CCK‐8 assay indicated that Huh‐7 and SMMC7721 cell survival were repressed by loss of circ_0008305 whereas induced by overexpression of circ_0008305. In addition, in Figure 2E,F, flow cytometry assay was carried out and it was shown that knockdown of circ_0008305 suppressed HCC cell apoptosis whereas cell apoptosis was reduced by up‐regulation of circ_0008305. In Figure 2G,H, it was proved that Huh‐7 and SMMC7721 cell migration were repressed by loss of circ_0008305. For another, transwell invasion assay was conducted and we found Huh‐7 and SMMC7721 cell invasion was remarkably reduced by the inhibition of circ_0008305 as displayed in Figure 2I,J. Oppositely, overexpression of circ_0008305 exhibited a reversed phenomenon.

### 3.3 Circ_0008305 sponged miR‐186 in HCC cells

**FIGURE 3:**
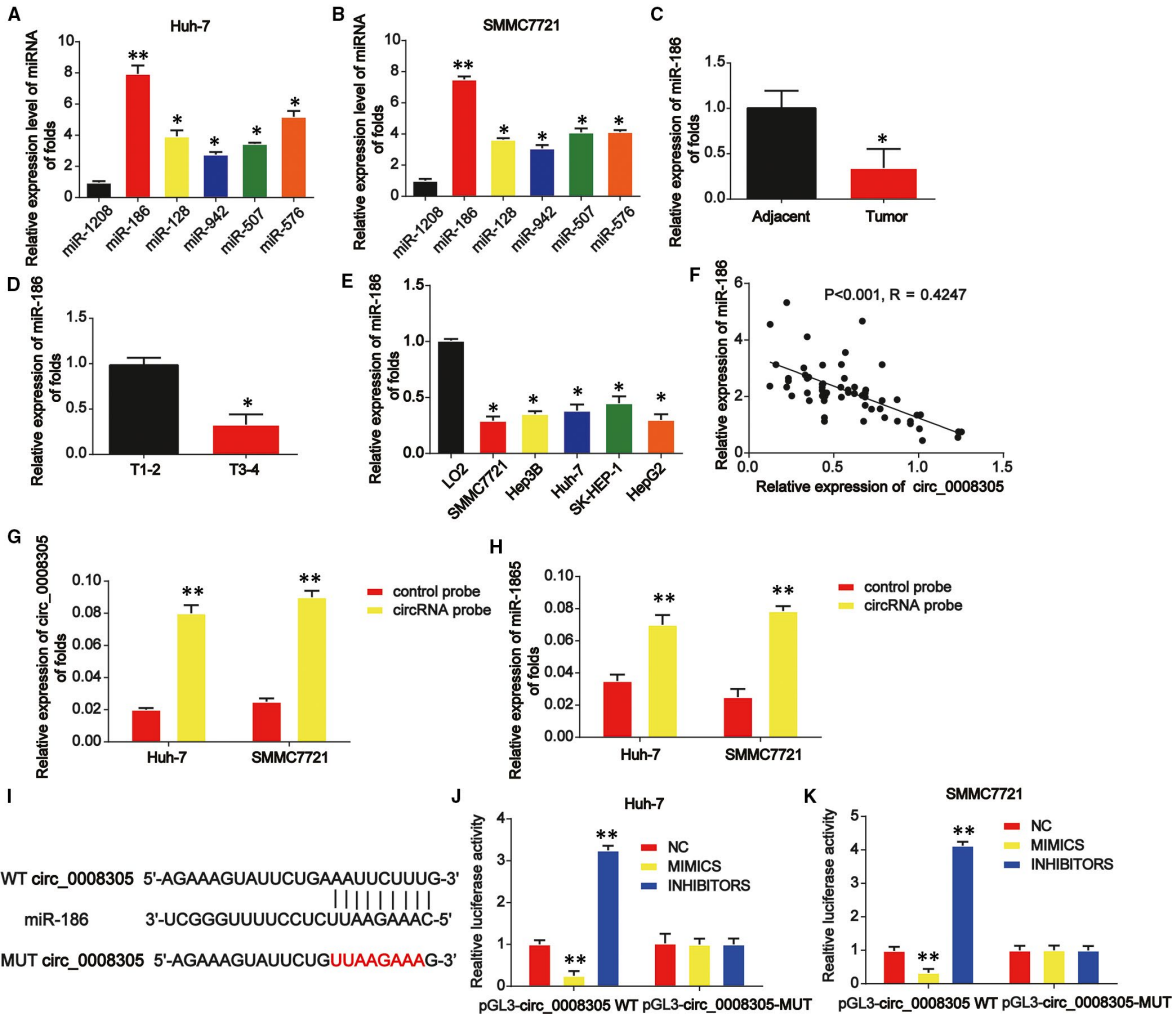
Circ_0008305 sponged miR‐186 in HCC cells. A and B, Six miRNA candidates in HCC cell lysates were detected by real‐time PCR. Multiple miRNAs were pulled down using circ_0008305 probe. C, The expression of miR‐186 in HCC carcinoma and normal adjacent tissues. U6 was used as internal control. D, Expression of miR‐186 in HCC tissues at different stages. E, The expression level of miR‐186 in HCC cells (SK‐HEP‐1, Huh‐7, Hep‐3B, HepG2 and SMMCC7721) and LO2 cells. F, The expression correlation between circ_0008305 and miR‐186 in HCC tissue samples. G and H, circ_0008305 and miR‐186 expression in the captured fraction of HCC cells by circ_0008305 probe. I, The putative binding sites between miR‐186 and circ_0008305. J and K, Luciferase activity was evaluated in HCC cells cotransfected with circ_0008305‐WT or circ_0008305‐MUT reporter and miR‐186 inhibitors, mimics or its scramble control (NC). Three independent experiments were carried out. Error bars stand for the mean ± SD of at least triplicate assays. *P < .05, **P < .01

Moreover, to find out whether circ_0008305 sponged microRNAs in HCC cells, a biotin‐labelled circ_0008305 probe was constructed. As displayed in Figure 3A,B, miR‐186 was most abundantly pulled down by circ_0008305 in Huh‐7 and SMMC7721 cells. Then, it was shown that miR‐186 was greatly reduced in HCC tissue samples in Figure 3C. In Figure 3D, miR‐186 expression was decreased in the HCC samples at T3‐T4 stage than in the HCC tissues at an early stage. As exhibited in Figure 3E, miR‐186 was greatly down‐regulated in HCC cells. A negative correlation between circ_0008305 and miR‐186 was observed in HCC tissue samples (Figure 3F). Additionally, an elevated enrichment of circ_0008305 and miR‐186 in the captured fraction of HCC cells by circ_0008305 probe was observed (Figure 3G,H). In addition, dual‐luciferase reporter assay proved that luciferase reporter plasmids of WT‐circ_0008305 and MUT‐circ_0008305 binding sites were shown in Figure 3I. cotransfection of the WT‐circ_0008305 with miR‐186 inhibitors increased the reporter activity while cotransfection with miR‐186 mimics repressed the reporter activity in HCC cells (Figure 3J,K).

### 3.4 Up‐regulation of miR‐186 depressed HCC cell growth, migration and invasion via targeting TMED2

**FIGURE 4:**
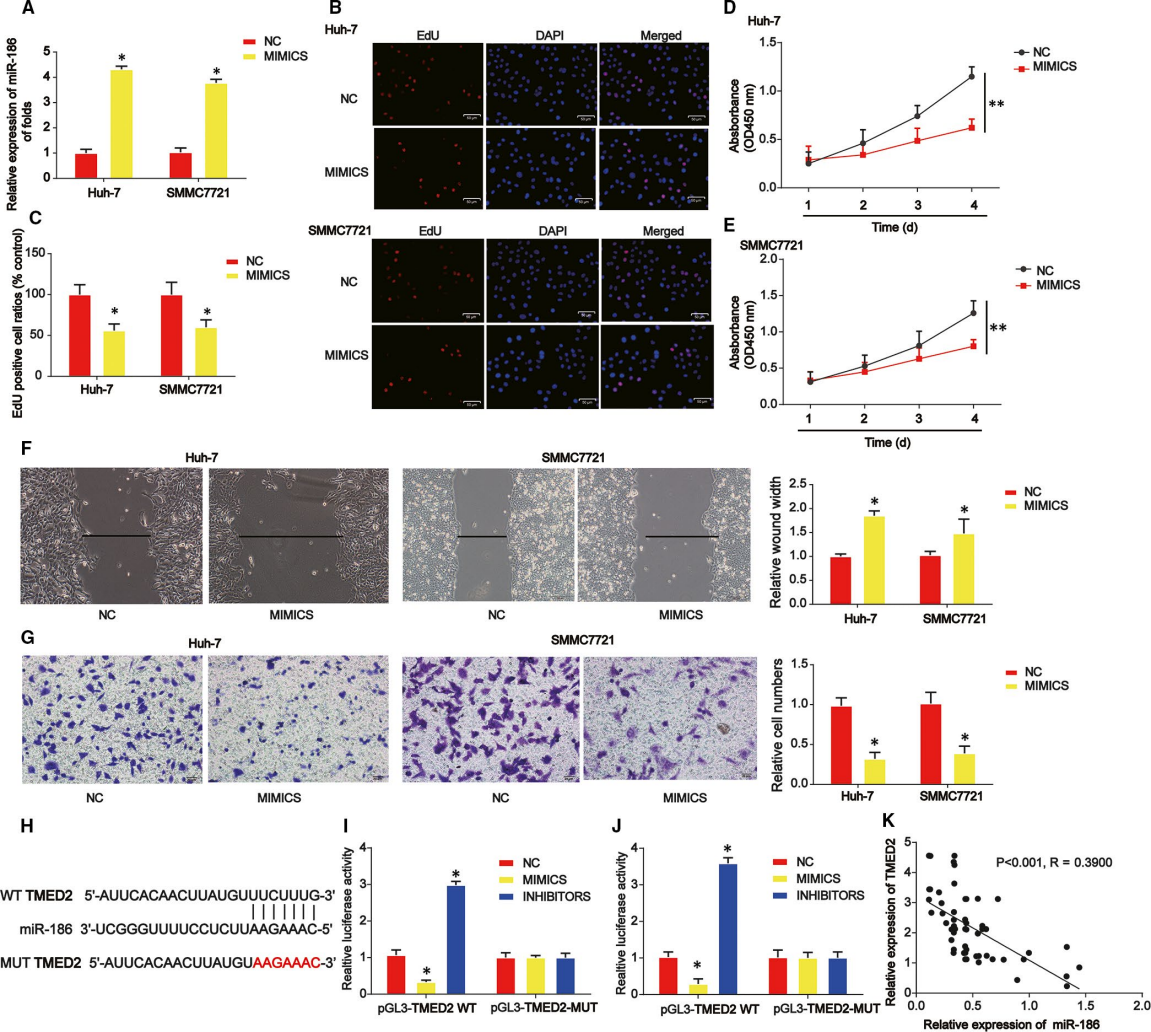
miR‐186 repressed HCC cell proliferation, migration and invasion via targeting TMED2. A, The expression of miR‐186 was detected by real‐time PCR in HCC cells. HCC cells were transfected with miR‐186 mimics. B and C, HCC cell proliferation was tested using EdU assay. D and E, HCC cell survival was evaluated using CCK‐8 assay. F, Effects of miR‐186 on HCC cell migration. Wound healing assay was carried out to detect cell migration capacity. G, Effects of miR‐186 on HCC cell invasion. Transwell invasion assay was used to detect cell invasion capacity. H, The putative binding sites between miR‐186 and TMED2. I and J, Luciferase activity was evaluated in HCC cells cotransfected with TMED2‐WT or TMED2‐MUT reporter and miR‐186 inhibitors, mimics or its scramble control (NC). K, The expression correlation between TMED2 and miR‐186 in HCC tissue samples. Three independent experiments were carried out. Error bars stand for the mean ± SD of at least triplicate assays. *P < .05

Then, we evaluated the effects and the potential mechanism of miR‐186 on HCC cell growth, migration and invasion. miR‐186 was significantly up‐regulated by miR‐186 mimics in Huh‐7 and SMMC7721 cells as demonstrated in Figure 4A. In Figure 4B,C, EdU assay proved that Huh‐7 and SMMC7721 cell proliferation was repressed by overexpression of miR‐186. In Figure 4D,E, HCC cell survival was also significantly reduced by miR‐186 as evidenced by CCK‐8 assay. In Figure 4F, Huh‐7 and SMMC7721 cell migration were reduced by increased miR‐186 as indicated using wound healing assay. In Figure 4G, transwell invasion assay displayed that Huh‐7 and SMMC7721 cell invasion were depressed by miR‐186 mimics. In addition, TMED2 was predicted as the target of miR‐186 using bioinformatics tools. Luciferase reporter plasmids of WT‐TMED2 and MUT‐TMED2 binding sites were exhibited in Figure 4H. In Figure 4I,J, cotransfection of the WT‐TMED2 with miR‐186 inhibitors induced the reporter activity while cotransfection with miR‐186 inhibitors enhanced the reporter activity in Huh‐7 and SMMC7721 cells. For another, we confirmed that there was a negative correlation between TMED2 and miR‐186 (Figure 4K).

### 3.5 The interaction between TMED2 and circ_0008305

**FIGURE 5:**
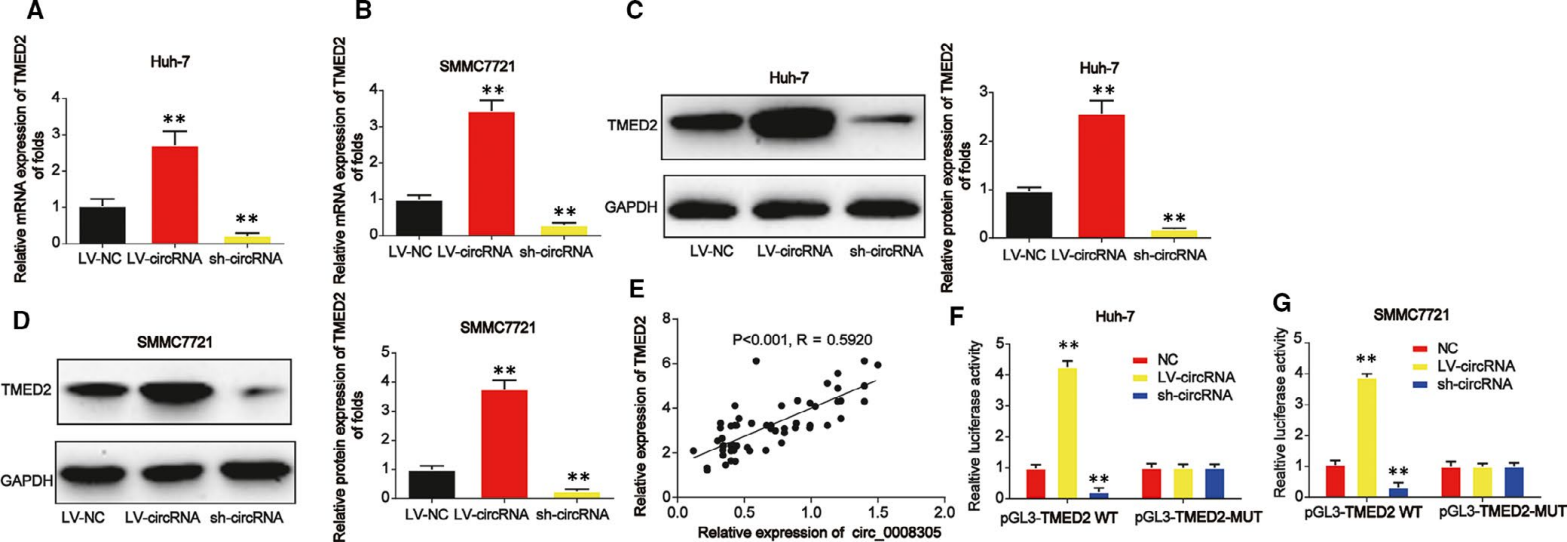
The correlation between circ_0008305 and TMED2. A and B, The mRNA expression of TMED2 was detected by real‐time PCR in HCC cells. HCC cells were transfected with TMED2 siRNA. C and D, The protein expression of TMED2 in HCC cells. E, The expression correlation between TMED2 and circ_0008305 in HCC tissue samples. F and G, Luciferase activity was evaluated in HCC cells cotransfected with TMED2‐WT or TMED2‐MUT reporter and LV‐circ_0008305 or circ_0008305 shRNA. Three independent experiments were carried out. Error bars stand for the mean ± SD of at least triplicate assays. **P < .01

Moreover, we assessed the correlation between TMED2 and circ_0008305. In Figure 5A‐D, TMED mRNA and protein expression were significantly induced by LV‐circ_0008305 while repressed by circ_0008305 shRNA in HCC cells. A positive association between TMED2 and circ_0008305 was validated in HCC tissue samples (Figure 5E). In addition, we proved that cotransfection of the WT‐TMED2 with LV‐circ_0008305 reduced the reporter activity while cotransfection with circ_0008305 shRNA promoted the reporter activity in Huh‐7 and SMMC7721 cells as manifested in Figure 5F,G.

### 3.6 Down‐regulation of TMED2 suppressed HCC cell growth, migration and invasion

**FIGURE 6:**
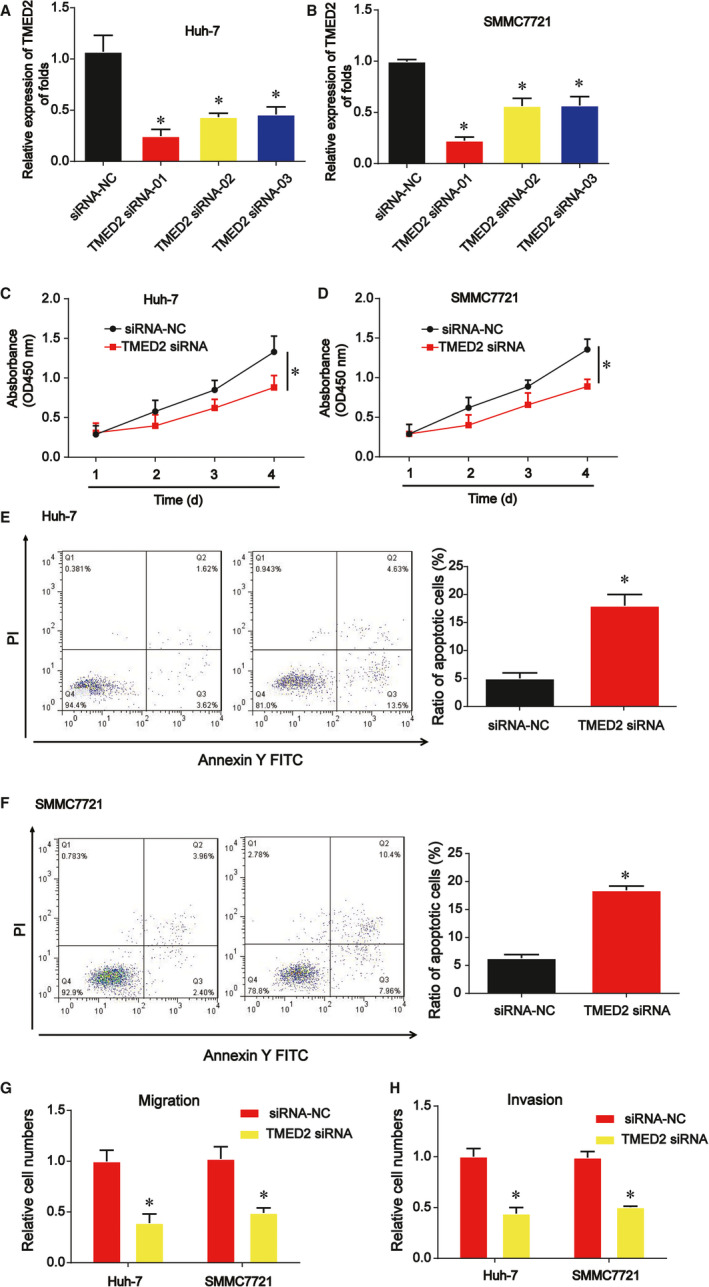
Effects of TMED2 on HCC cell proliferation, migration and invasion. A and B, The expression of TMED2 was detected by real‐time PCR in HCC cells. HCC cells were transfected with miR‐186 mimics. C and D, HCC cell survival was evaluated using CCK‐8 assay. E and F, Effects of TMED2 siRNA on HCC cell apoptosis. G and H, Effects of TMED2 siRNA on HCC cell migration and invasion. Three independent experiments were carried out. Error bars stand for the mean ± SD of at least triplicate assays. *P < .05

We focused the effects of TMED2 on HCC cell growth, migration and invasion. Firstly, we proved that TMED2 was significantly reduced by TMED2 siRNA in Huh‐7 and SMMC7721 cells in Figure 6A,B. siRNA‐01 of TMED2 was used for the subsequent assays. In Figure 6C,D, CCK‐8 assay proved that Huh‐7 and SMMC7721 cell survival were suppressed by loss of TMED2. In Figure 6E,F, we observed that HCC cell apoptosis was triggered by knockdown of TMED2. Besides these, we found that Huh‐7 and SMMC7721 cell migration and invasion were also greatly reduced by TMED2 down‐regulation as exhibited in Figure 6G,H.

### 3.7 Down‐regulated circ_0008305 suppressed the growth of HCC in vivo

**FIGURE 7:**
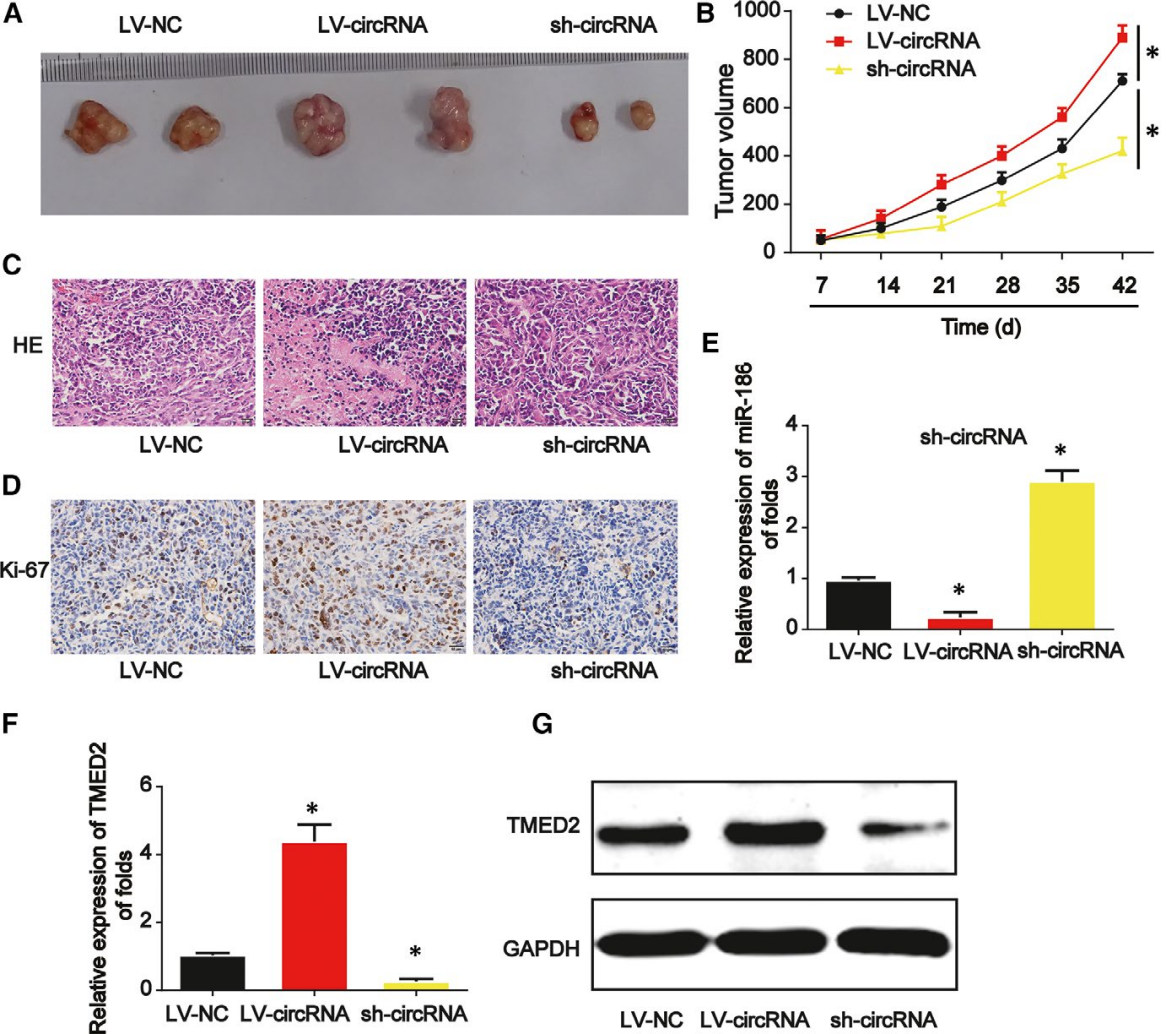
Down‐regulation of circ_0008305 repressed HCC progression through regulating miR‐186 and TMED2 in vivo. Twelve 8‐week old female BALB/c nude mice were injected with Huh‐7 cells infected with LV‐circ_0008305 or circ_0008305 shRNA. Six mice were used in each group. A, Tumours were peeled from the mice. B, Tumour volume. C, H＆E staining. D, IHC staining of Ki‐67 in tumour tissues. E, Expression of miR‐186. F, mRNA expression of TMED2. G, Protein expression of TMED2. Three independent experiments were carried out. Error bars stand for the mean ± SD of at least triplicate experiments. *P < .05

We confirmed the effects of circ_0008305 on tumour growth in vivo. Huh‐7 cells were transfected with circ_0008305 shRNA or LV‐circ_0008305 and then cells were injected into BALB/c nude mice subcutaneously. In Figure 7A, the tumours were peeled and exhibited. In Figure 7B, tumour volume was repressed by circ_0008305 shRNA while induced by LV‐circ_0008305 in a time‐dependent manner. Then, HE staining was demonstrated in Figure 7C and IHC staining of Ki‐67 implied circ_0008305 shRNA greatly reduced the tumour growth (Figure 7D). Finally, it was revealed that circ_0008305 functioned via regulating miR‐186 and TMED2 in vivo (Figure 7E,F,G).

## 4 DISCUSSION

In this work, we demonstrated an oncogenic role of circ_0008305 in the progression of HCC for the first time. The endogenous competitive relationships among circ_0008305/miR‐186/TMED2 were elaborated. We first reported that circ_0008305 was frequently up‐regulated. Then, we demonstrated that the overexpression of circ_0008305 induced the progression of HCC, while loss of circ_0008305 repressed HCC progression significantly. In addition, we revealed that circ_0008305 acted as a crucial ceRNA via competing for miR‐186. miR‐186 functioned as a downstream target for circ_0008305 and TMED2 served as a target for miR‐186. We observed that overexpression of miR‐186 restrained HCC progression via inhibiting TMED2.

Recently, the roles of circRNAs in cancers are attracting increasing attention.^15^, ^16^, ^17^ The molecular mechanisms and functions for circRNAs in these pathological processes still need to be elucidated clearly. Studies display that circRNAs can work as a miRNA sponge to protect mRNAs from attack of microRNAs.^18^, ^19^, ^20^ For example, CircSETD3 can inhibit HCC growth via sponging miR‐421.^21^ circ_0000204/miR‐191/KLF6 axis is involved in HCC cell proliferation.^22^ CircSLC3A2 has been identified as an oncogenic factor in HCC by sponging miR‐490‐3p and modulating PPM1F.^23^ Previously, circ_0008305 has been reported to repress lung cancer progression by controlling TIF1γ.^24^ In our current research, we performed qRT‐PCR and detected the expression levels of circ_0008305 in HCC tissues and HCC cells. The expression of circ_0008305 was elevated in HCC. Lack of circ_0008305 suppressed HCC progression whereas overexpression of circ_0008305 induced HCC development.

Then, circ_0008305 has been manifested to sponge miR‐186 using bioinformatics tools. Evidence has suggested the roles of miRNAs in carcinogenesis of human cancers. miR‐186 has been extensively investigated in various cancers.^25^ For instance, miR‐186‐5p can function as a tumour suppressor in osteosarcoma through targeting FOXK1.^26^ miR‐186 can promote tumour growth in cutaneous squamous cell carcinoma via repressing apoptotic protease activating factor‐1.^27^ In addition, miR‐186 can repress NSCLC migration via targeting cdc42.^28^ Currently, we investigated the function of miR‐186 in HCC. Increased miR‐186 exhibited an obvious tumour inhibitory role and it was confirmed as a target for circ_0008305.

TMED2 is an important member of transmembrane emp24 domain and TMED2 is involved in mouse embryos development.^29^, ^30^ TMED2 has been shown to participate in various cancers. For example, TMED2 can promotes epithelial ovarian cancer progression.^31^ Up‐regulated TMED2 is an unfavourable prognostic factor in breast cancer.^32^ Heterozygous mutation in TMED2 has been shown in non‐alcoholic fatty liver disease in mice.^33^ CircCDR1as modulate cell proliferation via regulating TMED2 and TMED10.^34^ However, the role of TMED2 in HCC progression is unknown. Here, in our work, TMED2 was predicted as a target for miR‐186. circ_0008305 was able to induce TMED2 expression via sponging miR‐186, thereby inhibiting HCC progression. Circ_0008305/miR‐186/TMED2 axis could be a potential prognostic biomarker of HCC. All of the above findings suggested that the circ_0008305/miR‐186/TMED2 axis played a significant role in HCC development.

We have demonstrated an oncogenic role of circ_0008305 in HCC progression for the first time. The competitive relationships among circ_0008305, miR‐186 and TMED2 were elaborated in our research. Our findings provided a platform for the potential mechanisms underlying HCC and identifying therapeutic targets.

## CONFLICT OF INTEREST

The authors confirm that there are no conflicts of interest.

## AUTHOR CONTRIBUTION

**xiaoyu zhang:** Data curation (equal); Software (equal); Writing‐original draft (equal). **Hui‐Hui Hao:** Data curation (equal); Investigation (equal); Visualization (equal). **Hai‐Wen Zhuang:** Data curation (equal); Formal analysis (equal); Software (equal). **Jian Wang:** Formal analysis (equal); Supervision (equal); Validation (equal). **Yu Sheng:** Investigation (equal); Methodology (equal); Visualization (equal). **Fang Xu:** Conceptualization (lead); Resources (lead); Writing‐review & editing (equal). **Jin Dou:** Project administration (equal); Validation (equal); Writing‐review & editing (equal). **Chuang Chen:** Data curation (equal); Investigation (equal); Visualization (equal). **Yang Shen:** Conceptualization (equal); Resources (equal); Writing‐review & editing (equal).

## Data Availability

The data that support the findings of this study are available from the corresponding author upon reasonable request.
